# O balanço hídrico pós-extubação se associa com falha da extubação: um estudo de coorte

**DOI:** 10.5935/0103-507X.20210057

**Published:** 2021

**Authors:** Priscila Albrecht dos Santos, Alexandre Ribas, Thiele Cabral Coelho Quadros, Clarissa Netto Blattner, Márcio Manozzo Boniatti

**Affiliations:** 1 Departamento de Terapia Intensiva, Hospital São Lucas, Pontifícia Universidade Católica do Rio Grande do Sul - Porto Alegre (RS), Brasil.; 2 Departamento de Terapia Intensiva, Hospital de Clínicas de Poto Alegre, Universidade Federal do Rio Grande do Sul - Porto Alegre (RS), Brasil.

**Keywords:** Equilíbrio hidroeletrolítico, Respiração artificial, Desmame, Extubação, Ventilação não invasiva

## Abstract

**Objetivo:**

Avaliar se há associação entre o balanço hídrico nas 48 horas após a extubação e a falha da extubação.

**Métodos:**

Este é um estudo de coorte prospectiva que incluiu os pacientes admitidos à unidade de terapia intensiva de um hospital terciário no sul do Brasil entre março e dezembro de 2019. Incluíram-se os pacientes que necessitaram de ventilação mecânica por pelo menos 24 horas e foram extubados durante o período do estudo. O desfecho primário foi falha da extubação, considerada como necessidade de reintubar dentro das primeiras 72 horas após a extubação. O desfecho secundário foi um desfecho combinado de falha da extubação ou necessidade de ventilação não invasiva terapêutica.

**Resultados:**

Foram incluídos 101 pacientes. Observou-se falha da extubação em 29 (28,7%) deles. Na análise univariada, pacientes com balanço hídrico negativo acima de 1L no período de 48 horas após a extubação tiveram menor taxa de falha da extubação (12,0%), em comparação a pacientes com balanço hídrico negativo nas 48 horas após a extubação menor que 1L (34,2%; p = 0,033). A duração da ventilação mecânica e o balanço hídrico negativo nas 48 horas após a extubação inferior a 1L se associaram com falha da extubação na análise multivariada quando corrigido pelo *Simplified Acute Physiology Score 3*. Quando avaliou-se o desfecho combinado, apenas o balanço hídrico nas 48 horas pós-extubação inferior a 1L manteve associação, quando corrigido pelo *Simplified Acute Physiology Score 3* e duração da ventilação mecânica.

**Conclusão:**

O balanço hídrico nas 48 horas após a extubação se associa com falha da extubação. São necessários mais estudos para avaliar se evitar um balanço hídrico positivo nesse período poderia melhorar os desfechos do desmame.

## INTRODUCTION

Failure of weaning from mechanical ventilation (MV), defined as failure of a spontaneous breathing test (SBT) or the need for tracheal reintubation within 48 - 72 hours following extubation, is associated with poor outcomes in critically ill patients.^(1)^ Specifically, the extubation failure rate varies from 10% - 20%,^(2-4)^ and it is important to determine which factors may be associated with this problem. Among the known causes, weaning-induced pulmonary edema is one of the most common.^(5-9)^ The transition from positive to negative intrathoracic pressure that occurs during weaning from MV can lead to cardiac dysfunction due to increased preload and afterload of the right and left ventricles, especially in patients with fluid overload.^(10)^ Even patients with normal cardiac function might develop alveolar transudation following extubation due to local changes in transpulmonary and capillary pressures.^(11-13)^

Several studies have demonstrated an association between pre-extubation fluid balance (FB) and extubation failure.^(2,3,14,15)^ The first randomized study to address fluid management during weaning was reported by Mekontso Dessap et al.^(16)^ Fluid management strategy guided by daily brain natriuretic peptide (BNP) plasma concentrations, with a significantly more negative FB, decreased the duration of weaning without increasing adverse consequences on hemodynamics or renal function. Recently, Liu et al. described the effects of fluid removal on the incidence of weaning-induced pulmonary edema.^(12)^ In this scenario, we hypothesize that the FB in the 48 hours after extubation, a little explored variable, can also be a risk factor for extubation failure. Thus, the aim of this study was to assess whether there is an association between 48-h postextubation FB and extubation failure.

## METHODS

This was a prospective cohort study that included patients admitted to the intensive care unit (ICU) of *Hospital São Lucas* (HSL) of the *Pontifícia Universidade Católica do Rio Grande do Sul* (PUC-RS) in Porto Alegre, Brazil, from March 2019 to December 2019. *Hospital São Lucas* is a tertiary hospital with 560 beds and approximately 26,000 hospitalizations per year. The ICU has 59 medical-surgical beds.

The study was approved by the HSL Research Ethics Committee. The Informed Consent form was signed by the patient or family member.

Patients who required MV for at least 24 hours and who were extubated during the study period were included. Patients with failure of previous extubation, whose 48-h postextubation FB was not recorded were excluded. The variables collected were age, sex, reason for starting MV, Simplified Acute Physiology Score 3 (SAPS 3), time on MV, use of noninvasive ventilation (NIV) after extubation, FB 24 hours and 48 hours pre-extubation and 48 hours postextubation, length of stay in the ICU and hospital and in-hospital mortality. Patients were followed up until hospital discharge.

Patients were considered ready for weaning from MV based on the following criteria: improvement of the underlying condition that led to respiratory failure, hemodynamic stability (mean arterial pressure of 65mmHg without or with minimal dose of vasoactive drugs), adequate level of consciousness without continuous sedation infusion, fraction of inspired oxygen (FiO_2_) < 50% and positive end-expiratory pressure (PEEP) ≤ 8cmH_2_O. A SBT was performed with a T-tube or pressure support ventilation (PSV) with 8cmH_2_O, both lasting 30 minutes. The decision to return the patient to MV or to perform extubation was based on signs of intolerance to SBT, such as tachypnea, tachycardia, hemodynamic instability, respiratory effort and altered state of consciousness. After extubation, the reintubation decision was based on the following criteria: decrease in oxygen saturation (SpO_2_) to < 88%, despite an increase in FiO_2_; decreased pH or increased partial pressure of carbon dioxide (PCO_2_); respiratory muscle fatigue; hemodynamic instability; copious secretion that the patient was unable to remove properly; and decreased level of consciousness. The use of NIV was recorded after extubation. Prophylactic NIV was defined as NIV initiated immediately after extubation, maintained for 4 hours and, after this period, used prophylactically. The use of NIV was defined as therapeutic when it was used to treat respiratory dysfunction within 72 hours after extubation. The use of therapeutic NIV was based mainly on tachypnea, a decrease in SpO_2_ or respiratory effort.

Fluid balance was defined as the total fluid input minus the total fluid output, without considering insensitive losses. Simple weaning was defined as extubation on the first SBT. The primary outcome was extubation failure, considered as the need for reintubation in the first 72 hours after extubation. The secondary outcome was a combined outcome with postextubation failure or the need for therapeutic NIV.

The sample was calculated considering an extubation failure rate of 15% and a mean difference of 1,500mL in 48-hour postextubation FB between patients with and without extubation failure, with a significance level of 5% and study power of 80%. The calculated sample size was 231 patients.

The statistical analysis of the collected data was performed through descriptive statistics with calculation of the mean ± standard deviation or median and interquartile range, as well as frequency and percentage. Statistical analyses were performed using the chi-square test to assess the associations between categorical variables and outcomes and the t-test or Mann-Whitney test to assess the associations between continuous variables and outcomes. All subjects were divided into categories according to FB in the 48 hours after extubation using arbitrary steps of 1,000mL as described by Frutos-Vivar et al.^(2)^ Subsequently, the 48-hour postextubation FB was dichotomized as < -1,000mL and > -1,000mL using the Youden Index (calculated as sensitivity plus specificity minus one). Finally, a logistic regression was performed. Variables with biological plausibility were chosen for the logistic regression model (SAPS 3, MV duration and 48-hour postextubation FB). As sensitivity analyses, we performed models with the 48-hour postextubation FB variable as ordinal and continuous. A value of p < 0.05 was considered statistically significant. Statistical analysis was performed using Statistical Package for the Social Sciences (SPSS) software version 20.0.

## RESULTS

During the study period, 112 patients were extubated after at least 24 hours of MV. Eleven patients were excluded for not having a FB record within 48 hours after extubation. Thus, 101 patients were included in the final analysis. Due to slow recruitment, the study had to be terminated prematurely. Demographic and clinical characteristics are described in table 1. Only four (3.9%) patients presented with congestive heart failure as a cause for MV.

**Table 1:** Demographic, clinical and outcome variables

	Success (n = 72)	Failure (n = 29)	p value
Age (years)	68.1 ± 15.5	59.2 ± 17.5	0.01
Sex (male)	44 (61.1)	15 (51.7)	0.39
SAPS 3	55.5 ± 24.1	51.3 ± 24.8	0.46
Cause for MV			0.33
COPD exacerbation	8 (11.1)	1 (3.4)	
Pneumonia	11 (15.3)	4 (13.8)	
Sepsis	13 (18.1)	9 (31.0)	
Congestive heart failure	3 (4.2)	1 (3.4)	
Neurological	14 (19.4)	10 (34.5)	
Postoperative	10 (13.9)	2 (6.9)	
Postcardiac arrest	4 (5.6)	0	
Hemodynamic instability	9 (12.5)	2 (6.9)	
SBT			0.76
T-tube	59 (81.9)	23 (79.3)	
PSV	13 (18.1)	6 (20.7)	
Duration of MV	5.0 (3.0 - 7.0)	6.0 (4.0 - 10.0)	0.16
Days of weaning	0 (0 - 1.0)	0 (0 - 1.0)	0.77
Ventilatory parameters before SBT			
PSV (cmH_2_O)	10.0 (10.0 - 12.0)	12.0 (10.0 - 12.0)	0.09
PEEP (cmH_2_O)	6.0 (6.0 - 7.0)	6.0 (5.5 - 7.0)	0.59
Tidal volume (mL)	486.9 ± 133.6	514.3 ± 142.8	0.36
FiO_2_	30.0 (25.0 - 35.0)	30.0 (30.0 - 35.0)	0.23
Vital signs before SBT			
Systolic arterial pressure (mmHg)	139.5 ± 27.2	136.2 ± 23.7	0.57'
HR (beats/minute)	93.4 ± 17.1	97.8 ± 18.4	0.25
RR (resp/minute)	19.9 ± 3.9	20.2 ± 5.3	0.77
SpO_2_	97.0 (96.0 - 99.0)	98.0 (95.0 - 99.0)	0.72
Fluid balance (mL)			
48-hour pre-extubation	668.9 ± 1,345.9	664.3 ± 1,291.4	0.99
24-hour pre-extubaion	158.0 ± 1,630.6	324.9 ± 1,787.7	0.66
48-hour postextubation	-173.3 ± 1,546.3	373.0 ± 1,102.5	0.09
Prophylactic NIV	19 (26.4)	5 (17.2)	0.441
Therapeutic NIV	13 (18.1)	12 (41.4)	0.01
ICU length of stay (days)	11.0 (8.0 - 16.0)	15.5 (13.0 - 31.0)	0.001
Hospital length of stay (days)	30.0 (19.25 - 47.5)	27.5 (16.25 - 55.25)	0.73
Tracheostomy	4 (5.6)	10 (34.5)	< 0.001
Hospital mortality	25 (34.7)	18 (62.1)	0.01

SAPS 3 - Simplified Acute Physiology Score 3; MV - mechanical ventilation; COPD - chronic obstructive pulmonary disease; SBT - spontaneous breathing test; PSV - pressure support ventilation; PEEP - positive end-expiratory pressure; FiO_2_ - fraction of inspired oxygen; HR - heart rate; RR - respiratory rate; SpO_2_ - oxygen saturation; NIV - noninvasive ventilation; ICU - intensive care unit. Results expressed as the mean ± standard deviation, n (%) or median (interquartile range).

Extubation failure was observed in 29 (28.7%) patients. Patients with extubation failure were younger and had a longer ICU stay, greater need for tracheostomy and higher hospital mortality. Therapeutic NIV was used in 25 (24.7%) patients and was more common in patients with extubation failure.

**Figure 1:**
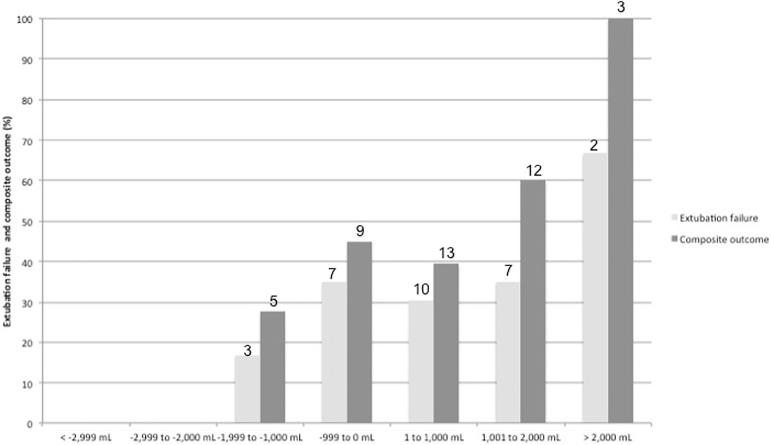
Prevalence of extubation failure by fluid balance category. Subjects were divided into categories using arbitrary steps of 1,000mL.

Using arbitrary steps of 1,000mL, the prevalence of extubation failure was higher in patients with positive FB (Figure 1). Patients with a negative 48-hour postextubation FB higher than one liter had a lower rate of extubation failure (12.0% *versus* 34.2%; p = 0.033) and combined outcome (20.0% *versus* 48.7%; p = 0.012). Mechanical ventilation duration and 48-hour postextubation FB were associated with extubation failure when corrected for SAPS 3 in multivariate analysis (Table 2 and Tables 1S - 4S in Supplementary material). When we evaluated the combined outcome, only 48-hour postextubation FB maintained an association when corrected for SAPS 3 and MV duration.

**Table 2:** Multivariate analysis for extubation failure and the combined outcome

	Extubation failure	Combined outcome
	Adjusted OR	Adjusted OR
Negative 48-h postextubation FB lower than 1L	4,528 (1,165 - 17,595)	4,151 (1,364 - 12,633)

Model adjusted on Simplified Acute Physiology Score 3 and mechanical ventilation duration. OR - odds ratio; FB - fluid balance.

## DISCUSSION

We found an association between FB in the 48 hours after extubation and extubation failure. This is the first study that demonstrates the association between this outcome and FB after extubation, instead of FB in the hours before extubation, a variable traditionally investigated.

There is a strong biological plausibility for the FB to be associated with weaning or extubation failure. A positive FB can lead to capillary leakage, with an increase in pulmonary extravascular water, contributing to respiratory dysfunction after extubation.^(17)^ In addition, cardiovascular dysfunction is increasingly recognized as an important cause of weaning failure, even in patients without previously recognized heart disease.^(18)^ The incidence of weaning-induced cardiac dysfunction as a cause of failure in this process varies from 20% to 87%.^(5-9)^ When the patient resumes spontaneous breathing, he/she restores negative values of inspiratory intrathoracic pressures, thus increasing venous return, central blood volume and left ventricular afterload.^(19)^ In this scenario, volume overload can contribute to a decompensation in cardiorespiratory function. In our study, we verified an association of FB with extubation failure, even in a population with a low prevalence of chronic obstructive pulmonary disease or congestive heart failure, and with a predominance of simple weaning. Our hypothesis is that hypervolemia can contribute to extubation failure independent of the primary cause. Patients who are fluid overloaded are more prone to fail extubation than euvolemic patients, even if the primary cause was not identified as congestive heart failure. This hypothesis must be confirmed in larger studies.

Several previous studies have found an association between pre-extubation FB, both 24 hours and cumulative balance, and extubation failure.^(3,14,15,20)^ We did not verify this association with pre-extubation FB, similar to a previous study that evaluated the outcome of weaning failure.^(21)^ One possible explanation is that the pre-extubation FB is already routinely assessed, which may interfere with the decision to extubate the patient.

With the frequent use of therapeutic NIV after extubation,^(22)^ the combined outcome (reintubation or use of therapeutic NIV) is possibly more sensitive to identify patients with significant respiratory dysfunction after extubation. Fluid balance after extubation maintained its association with the combined outcome.

In relation to fluid interventions during weaning, Mekontso Dessap et al. found that pre-extubation fluid guided by BNP values resulted in a shorter duration of MV.^(16)^ Other authors have suggested randomized studies to assess the role of diuretic therapy in preventing extubation failure.^(2,3)^ Our data suggest that this investigation should be extended to at least 48 hours after extubation.

Younger patients had a higher rate of extubation failure, which is the opposite of that previously verified in the literature.^(23)^ However, when we added the use of therapeutic NIV, age was not associated with the combined outcome. Older patients used more NIV, which may have changed the outcome of extubation failure within 72 hours. Another point to be highlighted is that our reintubation rate was higher than most previous studies.

Our study has some limitations. First, it is an observational study, and it is not possible to demonstrate a causal relationship between FB and extubation failure. Second, we did not reach the estimated sample size, which may compromise the power of the study for the conclusions presented. Third, the number of patients included was small, in addition to being a single-center study, which makes it difficult to generalize the results. Fourth, we did not collect hemodynamic or echocardiographic measurements to correlate positive FB with ventricular dysfunction. Finally, we did not determine the cause of reintubation.

## CONCLUSION

We found that 48-h postextubation fluid balance was associated with extubation failure. Further studies are necessary to assess whether avoiding positive fluid balance in this period might improve weaning outcomes.
