# Supplementary material for: O balanço hídrico pós-extubação se associa com falha da extubação: um estudo de coorte
Source: Rev Bras Ter Intensiva. 2021 Jul-Sep;33(3):422–7. doi: 10.5935/0103-507X.20210057 (PMC8555406; doi:10.5935/0103-507X.20210057)
Supplement: Supplementary file 1 [file rbti-33-03-0422-suppl01.pdf]

Postextubation fluid balance is associated with extubation failure: a cohort study

O balanço hídrico pós-extubação se associa com falha da extubação: um estudo de coorte

Priscila Albrecht dos Santos[1], Alexandre Ribas[1], Thiele Cabral Coelho Quadros[1], Clarissa Netto Blattner[1], Márcio Manozzo Boniatti[2]

Table 1S - Multivariate analysis for extubation failure with 48-h postextubation fluid balance as a continuous variable

	OR	95%CI	p value
48-h postextubation FB	1,000	1,000 – 1,001	0.075

Model adjusted on Simplified Acute Physiology Score 3 and mechanical ventilation duration. OR - odds ratio; 95%CI - 95% confidence interval; FB - fluid balance.

Table 2S - Multivariate analysis for combined outcome with 48-h postextubation fluid balance as a continuous variable

	OR	95%CI	p value
48-h postextubation FB	1,001	1,000 - 1,001	0.004

Model adjusted on Simplified Acute Physiology Score 3 and mechanical ventilation duration. OR - odds ratio; 95%CI - 95% confidence interval; FB - fluid balance.

Table 3S - Multivariate analysis for extubation failure with 48-h postextubation fluid balance as an ordinal variable

48-h postextubation FB	OR	95%CI	p value
≤ -1,000			
-999 to 0	4,750	0.965 - 23,378	0.055
1 to 1,000	3,651	0.836 - 15,933	0.085
> 1,000	5,944	1,273 - 27,745	0.023

Model adjusted on Simplified Acute Physiology Score 3 and mechanical ventilation duration. OR - odds ratio; 95%CI - 95% confidence interval; FB - fluid balance.

Tabela 4S - Multivariate analysis for combined outcome with 48-h postextubation fluid balance as an ordinal variable

48-h postextubation FB	OR	95%CI	p value
≤ -1,000	Reference		
-999 - 0	3,508	0.895 - 13,748	0.072
1 - 1,000	2,788	0.809 - 9,605	0.809 - 9,605
> 1,000	8,804	2,282 - 33,972	0.002

Model adjusted on Simplified Acute Physiology Score 3 and mechanical ventilation duration. OR - odds ratio; 95%CI - 95% confidence interval; FB - fluid balance.
